# Chaperone Proteins in the Central Nervous System and Peripheral Nervous System after Nerve Injury

**DOI:** 10.3389/fnins.2017.00079

**Published:** 2017-02-21

**Authors:** Shalina S. Ousman, Ariana Frederick, Erin-Mai F. Lim

**Affiliations:** ^1^Departments of Clinical Neurosciences and Cell Biology & Anatomy, Hotchkiss Brain Institute, University of CalgaryCalgary, AB, Canada; ^2^Department of Neuroscience, Hotchkiss Brain Institute, University of CalgaryCalgary, AB, Canada

**Keywords:** peripheral nerve injury, spinal cord injury, axotomy, chaperones, chaperone proteins, regeneration, neuronal cell death, myelination

## Abstract

Injury to axons of the central nervous system (CNS) and the peripheral nervous system (PNS) is accompanied by the upregulation and downregulation of numerous molecules that are involved in mediating nerve repair, or in augmentation of the original damage. Promoting the functions of beneficial factors while reducing the properties of injurious agents determines whether regeneration and functional recovery ensues. A number of chaperone proteins display reduced or increased expression following CNS and PNS damage (crush, transection, contusion) where their roles have generally been found to be protective. For example, chaperones are involved in mediating survival of damaged neurons, promoting axon regeneration and remyelination and, improving behavioral outcomes. We review here the various chaperone proteins that are involved after nervous system axonal damage, the functions that they impact in the CNS and PNS, and the possible mechanisms by which they act.

## PNS and CNS nerve damage

**Figure 1:**
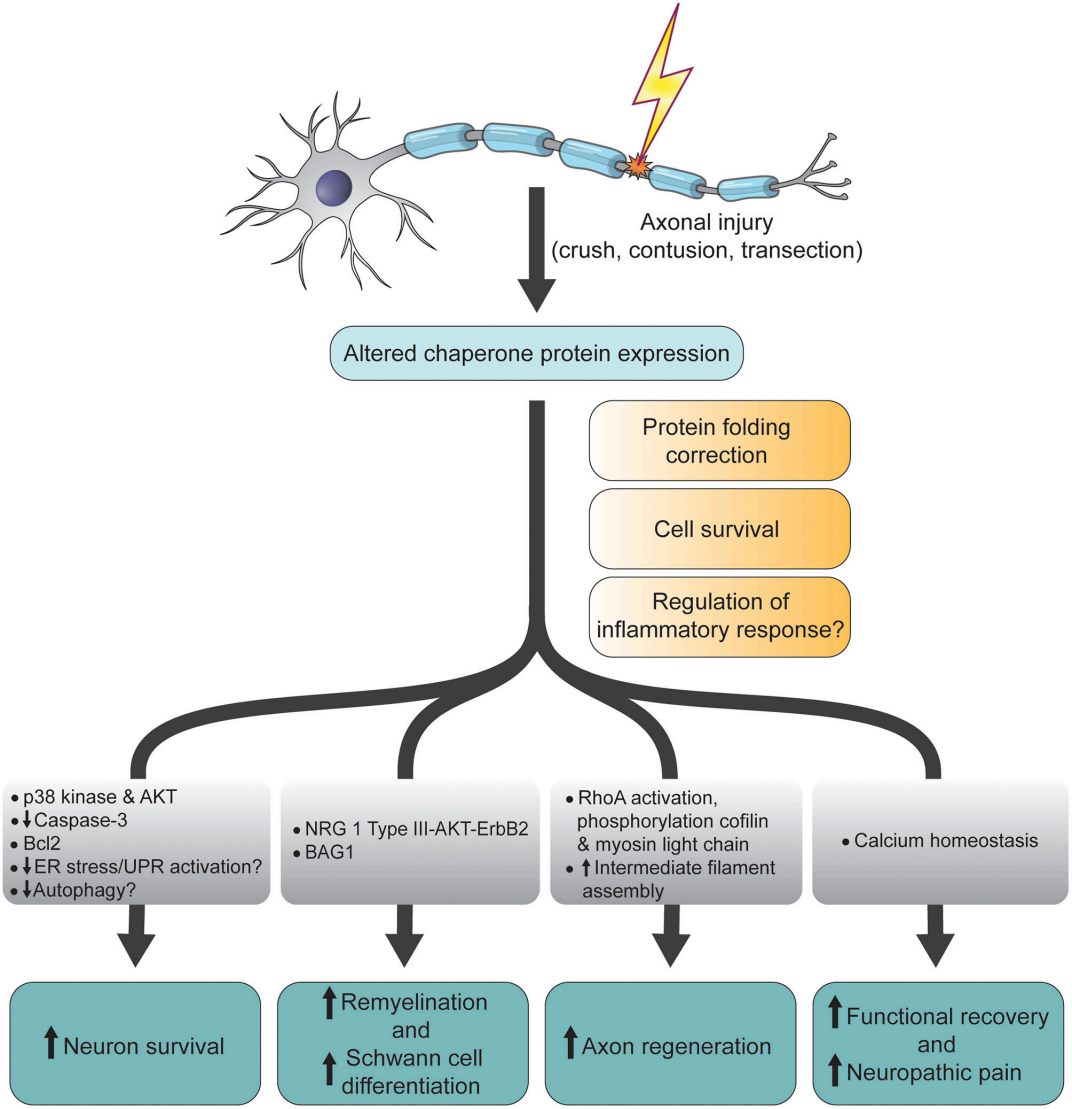
**Schematic diagram summarizing the functions and molecular changes associated with altered chaperone protein expression following CNS and PNS axonal injury**. ER, endoplamic reticulum; UPR, unfolded protein response; NRG, neuregulin; Bcl2, B-cell lymphoma 2; BAG1, Bcl-2-associated athanogene-1; AKT, AKT8 virus oncogene cellular homolog; p38, p38 mitogen-activated protein kinase.

Following damage to the axons of central nervous system (CNS) and peripheral nervous system (PNS) neurons, a number of cellular and molecular processes are initiated to promote regeneration of damaged nerve fibers, remyelination and target reinnervation (Zochodne, 2008). In the injured PNS for example, re-expression of regeneration associated genes (RAGs) such as c-jun and growth associated protein-43, are involved in axon outgrowth (Hall, 2005; Zochodne, 2008) while inhibitory axonal and myelin debris is phagocytosed by Schwann cells and infiltrating hematogenously-derived macrophages (Waller, 1850; Gaudet et al., 2011). Schwann cells also (Liu H. M. et al., 1995) secrete neutrophic factors (Madduri and Gander, 2010) and adhesion molecules (Colognato et al., 2005; Nodari et al., 2008) that allow for survival and directional growth of regenerating axons. Because of these favorable conditions, axotomized PNS neurons generally regenerate robustly, although less so in humans. Compared to the PNS however, injured CNS neurons regrow poorly and this has been attributed to reduced and/or premature truncation of beneficial processes. For example, the damaged CNS displays inadequate RAG expression, poor immune responses and, death of oligodendrocytes (David and Ousman, 2002). As a consequence, many labs are interested in identifying the molecular factors that promote axon regeneration in the damaged CNS and PNS. Over the last three decades, it has become evident that chaperone proteins are involved in the PNS and CNS after nerve damage. This mini-review will focus on which chaperones have been found to be modulated following axon damage (Table 1) and, the functions that they have been assigned (Figure 1).

**Table 1:** **Expression of chaperone proteins after CNS and PNS axotomy**.

**Protein**	**PNS/CNS, species**	**Tissue**	**Cell type**	**Change**	**Authors**
alphaB-crystallin	CNS, mouse	Spinal cord	Astrocytes and oligodendrocytes	Decreased	Klopstein et al., 2012
	PNS, rat	DRG	Neurons	Increased	Willis et al., 2005
	PNS, mouse	Sciatic nerve	Schwann cells and neurons	Decreased	Lim et al., 2017
BAG1	PNS, rat	Sciatic nerve	Schwann cells	Increased	Wu et al., 2013
BiP	CNS, rat	Spinal cord	Neurons and glia	Unchanged	Penas et al., 2007
	CNS, rat	Spinal cord	Neurons and glia	Decreased	Penas et al., 2011a
Clusterin	CNS, rat	Hippocampus	Astrocytes and neurons	Increased	Lampert-Etchells et al., 1991
	CNS, rat	Brain	Non-neuronal	Increased	Zoli et al., 1993
	CNS, rat	Spinal cord	Astrocytes and oligodendrocytes	Increased	Liu et al., 1998
	CNS, rat	Spinal cord	Glia	Increased	Liu L. et al., 1995
	CNS, rat	Red nucleus	Neurons and glia	Increased	Liu et al., 1999
	CNS, rat	Spinal cord	Neurons and glia	Increased	Klimaschewski et al., 2001
	CNS, rat	Spinal cord and brain	Neurons and glia	Increased	Törnqvist et al., 1996
	PNS, rat	Sciatic nerve	Non-specific	Increased	Bonnard et al., 1997
	PNS, rat	Sciatic nerve	Schwann cells	Increased	Wright et al., 2014
	PNS, rat	Hypoglossal nerve	Neurons and glia	Increased	Törnqvist et al., 1996
	PNS, rat	Hypoglossal nerve	Neurons and glia	Increased	Svensson et al., 1995
GRP75	PNS, rat	DRG	Neurons	Increased	Willis et al., 2005
GRP94	CNS, rat	Spinal cord	Neurons and astrocytes	Increased	Xu et al., 2011
HSP25	CNS and PNS, mouse	Spinal cord and sciatic nerve	Neurons	Increased	Murashov et al., 2001
	PNS, rat	Inferior alveolar nerve	Schwann cells, neurons, and endothelial cells	Increased	Iijima et al., 2003
HSP27	CNS, hamster	Retina	Retinal ganglion cells	Increased	Liu et al., 2013
	CNS, mouse	Spinal cord	n/a	Increased	Yi et al., 2008
	CNS, rat	Spinal cord	n/a	Increased	Zhang et al., 2010
	CNS, rat	Spinal cord	n/a	Increased	Park et al., 2007
	CNS, rat	Spinal cord	Neurons	Increased	Keeler et al., 2012
	CNS, rat	Brainstem	Neurons	Increased	Vinit et al., 2011
	CNS, rat	Retina	Retinal ganglion cells	Increased	Hebb et al., 2006
	CNS, rat	Retina, optic tract, and superior colliculus	Retinal ganglion cells and astrocytes	Increased	Krueger-Naug et al., 2002
	CNS, rat	Medulla oblongata	Neurons	Increased	Hopkins et al., 1998
	CNS, rat	Retina, optic nerve, optic tract, lateral geniculate leaflet, visual cortex	Non-specific; astrocytes	Increased	Chidlow et al., 2014
	CNS and PNS, rat	Sciatic nerve, DRG, spinal cord	Neurons	Increased w/anterograde transport	Costigan et al., 1998
	PNS, rat	Sciatic nerve	Schwann cells	Increased	Hirata et al., 2003
	PNS, rat	DRG	Neurons	Increased	Benn et al., 2002
	PNS, rat	Sciatic nerve	Non-specific	Increased	Klass et al., 2008
	PNS, rat	Sciatic nerve	Non-specific	Increased	Kim et al., 2001
	PNS, rat	Sciatic nerve	n/a	Increased	Tsubouchi et al., 2009
HSP30	CNS, goldfish	Retina	n/a	Increased	Cauley et al., 1986
HSP32	CNS, mouse	Spinal cord	n/a	Increased	Yi et al., 2008
	CNS, rat	Spinal cord	n/a	Increased	Park et al., 2007
HSP60	PNS, rat	DRG	Neurons	Increased	Willis et al., 2005
HSP70	CNS, zebrafish	Optic nerve	Retinal ganglion cells	Increased	Nagashima et al., 2011
	CNS, rabbit	Spinal cord	Neurons	Increased	Sakurai et al., 1997
	CNS, mouse	Spinal cord	n/a	Increased	Yi et al., 2008
	CNS, rat	Spinal cord	Neurons and glia	Increased	Gower et al., 1989
	CNS, rat	Spinal cord	Macrophages and glia	Increased	Mautes and Noble, 2000; Mautes et al., 2000
	CNS, rat	Spinal cord	n/a	Increased	Park et al., 2007
	CNS, rat	Spinal cord	n/a	Increased	Sengul et al., 2013
	CNS, rat	Spinal cord	n/a	Increased	Zhang et al., 2010
	CNS, rat	Spinal cord	Non-specific	Increased	Song et al., 2001
	CNS, rat	Spinal cord	Neurons	Increased	Keeler et al., 2012
	CNS, rat	Spinal cord	Non-specific	Increased	Kalmar et al., 2003
	CNS, rat	Retina, optic nerve, optic tract, lateral geniculate leaflet, visual cortex	Non-specific	No change	Chidlow et al., 2014
	CNS & PNS, dog	Spinal cord and DRG	Neurons and glia	Increased	Cízková et al., 2005
	PNS, hamster	Facial nerve	Non-specific	Increased	New et al., 1989
	PNS, frog	Sciatic nerve	Neurons	Retrograde transport	Edbladh et al., 1994
	PNS, rat	Sciatic nerve	Non-specific	Increased	Klass et al., 2008
HSP72	CNS, rat	Spinal cord	n/a	Increased	Sharma et al., 2015
	CNS, rat	Spinal cord	Neurons	Increased	Sharma et al., 2006
	CNS, rat	Spinal cord	Non-specific	Increased	Tachibana et al., 2002
	CNS, rat	Spinal cord	n/a	Increased	Chang et al., 2014
	PNS, rat	External carotid nerve ganglion	Schwann cells and neurons	Increased	Hou et al., 1998
HSP90	CNS, rat	Spinal cord	n/a	Increased nitration	Franco et al., 2013
	PNS, rat	Sciatic nerve	Non-specific	Increased	Klass et al., 2008
HSP90ab1	CNS, rat	Spinal cord	Non-specific	Decreased	Zhou et al., 2014
HSPa4	CNS, rat	Spinal cord	Non-specific	Decreased	Zhou et al., 2014
HSPe1	CNS, rat	Spinal cord	Non-specific	Decreased	Zhou et al., 2014
σ1R	PNS, rat	DRG	Neurons and satellite glia	Decreased	Bangaru et al., 2013

## Chaperone proteins

There are thousands of original manuscripts and reviews on chaperone proteins and so we will only introduce them in general terms here. Proteins have to be in a specific conformation in order to perform their functions. When cells experience stresses such as high and low temperatures, altered pH, oxygen deprivation, or disease states, proteins have difficulty in forming and maintaining their proper structures. Also, misfolded proteins can cause correctly structured ones to unfold. If not corrected, misfolded proteins can form aggregates that could lead to cell death (Reddy et al., 2008). Chaperones assist in the correct non-covalent assembly of polypeptides. They have the ability to recognize unfolded or partially denatured proteins and prevent incorrect associations and aggregation of unfolded polypeptide chains (Derham and Harding, 1999). In addition to correcting protein misfolding, some chaperones such as heat shock protein (HSP)70 and alphaB-crystallin (αBC)/HSPB5 promote (Campisi and Fleshner, 2003) or suppress (Ousman et al., 2007) inflammatory responses, while others such as HSP27 and αBC are involved in cell survival (Parcellier et al., 2005). Chaperones therefore play an important role in maintaining cell homeostasis and survival.

## Expression of chaperones after PNS and CNS nerve injury

Hsps were one of the earliest chaperones discovered to have altered expression after PNS and CNS axonal damage. Cauley et al. (1986) found that expression of a 30 kDa HSP was augmented after optic nerve crush in goldfish. HSP70 was subsequently discovered to be induced after facial nerve axotomy in hamsters (New et al., 1989) and following crush damage to rat spinal cords (Gower et al., 1989), along with the ability to be retrogradely transported in damaged frog sciatic nerves (Edbladh et al., 1994). Other groups have also observed augmented expression of HSP70 in transected zebrafish optic nerves (Nagashima et al., 2011) and rat spinal cord (Keeler et al., 2012) as well as in macrophages and astrocytes after spinal cord contusion in rats (Mautes and Noble, 2000). After these first observations, increased expression of other hsps including HSP25 (Iijima et al., 2003) and HSP27 (Hirata et al., 2003) has been noted in the retina, optic tract and superior colliculus of transected rat optic nerves (Krueger-Naug et al., 2002; Hebb et al., 2006), in transected rat spinal cord (Keeler et al., 2012), and in inferior alveolar and sciatic nerves and their associated Schwann cells and dorsal root ganglia (DRG) (Costigan et al., 1998; Kim et al., 2001). Of interest, there may be some selectivity in the location of HSP27 and phosphorylated-HSP27 after cervical spinal cord injury since the hsp was found to be expressed only in subpopulations of injured neurons in the rostral ventral respiratory group, the dorsal part of the gigantocellularis (Gi), and vestibular nucleus, but seldom in the ventral Gi and raphe nucleus (Vinit et al., 2011). Work by Willis et al. (2005) expanded the list of HSPs found to be enhanced in the injured PNS to include HSP60, glucose-regulated protein (GRP)75 and αBC which they discovered were capable of being synthesized within damaged PNS axons. In the CNS, increases in the levels of HSP32, HSP72 and HSP90 were evident following transection of the spinal cord and even after constriction and transection of peripheral nerves (Klass et al., 2008; Sharma et al., 2015). The latter observation showed that PNS nerve damage could alter expression of hsps in the CNS.

Other chaperone proteins besides hsps have been associated with axonal damage in the PNS and CNS. In 1997, Bonnard et al. (1997) found that clusterin/ApoJ/sulfated glycoprotein-2 mRNA increased in rat sciatic nerve after crush damage with expression of the chaperone possibly being specific to sensory axons as observed in a mouse sciatic nerve transection paradigm (Wright et al., 2014). Since the Bonnard observation, an augmentation in clusterin mRNA and protein has also been seen in numerous nerve injury scenarios including the spinal cord dorsal horn and gracile nucleus following rat sciatic nerve transection (Liu L. et al., 1995), the rat hippocampus after entorhinal cortex lesioning (Lampert-Etchells et al., 1991), the mesodiencephalic after hemitransection (Zoli et al., 1993), and the hypoglossal nucleus after hypoglossal nerve transection (Svensson et al., 1995). Finally, GRP94 is another chaperone whose expression increases after contusion injury in the rat spinal cord (Xu et al., 2011) while Bcl-2-associated athanogene-1 (BAG1), a co-chaperone for HSP70/HSC70 expression, is enhanced in Schwann cells following sciatic nerve crush in rats (Wu et al., 2013).

Not all chaperones however are augmented after PNS and CNS injury. Klopstein et al. (2012) noted a reduction in the small hsp, αBC, after spinal cord contusion injury in mice, and we have also observed a decrease in this crystallin after sciatic nerve crush damage in mice (Lim et al., 2017). Further, sigma-1 receptor (σ1R), an endoplasmic reticulum chaperone protein which is present in both sensory neurons and satellite cells in rat DRGs, was found to be downregulated in neurons as well as in their accompanying satellite glial cells after sciatic nerve ligation (Bangaru et al., 2013). HSP60 is another chaperone that was decreased in the brains of rats with pain and motor deficits following sciatic nerve ligation (Mor et al., 2011) while HSP90ab1, HSPa4, and HSPe1 were reduced after contusion injury in rats (Zhou et al., 2014).

Altogether then, the expression of various chaperones and co-chaperones is altered after CNS and PNS axon damage in either an enhanced or reduced manner. The question is, do these proteins play an active role in the injury or repair processes.

## Function of chaperone proteins after PNS and CNS axonal damage

### Neuronal survival

Because of the increased expression of clusterin in axotomized motorneurons, Törnqvist et al. (1996) suggested that the chaperone may be involved in the death of damaged neurons. Other studies instead indicate that some chaperone proteins play a role in maintaining survival of damaged neurons. For instance, Benn et al. (2002) found that upregulation and phosphorylation of HSP27 was required for the survival of sensory and motor neurons after sciatic nerve transection. The lab then extended this work both *in vivo* and *in vitro* to show that survival of PNS neurons from injured neonatal or adult animals following nerve growth factor (NGF) removal was related to whether DRG cells expressed HSP27. They also demonstrated that overexpression of human HSP27 in neonatal rat sensory and sympathetic neurons significantly increased survival after NGF withdrawal (Lewis et al., 1999). With respect to other chaperone proteins, HSP70 was observed to promote retinal ganglion cell survival and optic nerve regeneration in zebrafish since these processes were enhanced and reduced respectively if the hsp was inhibited (Nagashima et al., 2011). Moreover, exogenous application of HSP70 to the proximal end of transected rat sciatic nerves prevented the death of almost all sensory neurons (Houenou et al., 1996). Other evidence for a role of chaperones in promoting neuronal survival after CNS and PNS injury was demonstrated by Chang et al. (2014) who showed that HSP72 expression in neurons and astrocytes correlated with less apoptosis of these cell types in a rat spinal cord compression model. Also, survival of retinal ganglion cells following optic nerve transection in hamsters correlated with HSP27 expression after remote ischemic pre-conditioning (Liu et al., 2013) while Penas et al. (2011a) showed that a decrease in binding immunoglobulin protein (BiP)/GRP78 correlated with retrograde degeneration of damage peripheral motor neurons. Interestingly, if chaperones themselves are altered by post-translational modification, this could negatively impact neuronal survival as shown by Franco et al. (2013) who found that the presence of nitrated HSP90 in contused rat spinal cord was linked to motor neuron death.

With respect to which molecular mechanism(s) may be involved in chaperone-mediated neuronal survival post-nerve damage, the activation of the p38 kinase was found to be required for induction of HSP25 expression after sciatic nerve axotomy. Here, HSP25 formed a complex with AKT to prevent neuronal cell death (Murashov et al., 2001). Also, nitrated HSP90 induced neuronal death via P2X7 receptor-dependent activation of the Fas pathway (Franco et al., 2013) while HSP27's neuroprotective action was found to be downstream of cytochrome c release from mitochondria and upstream of caspase-3 activation (Benn et al., 2002). Another possible mechanism involved in cell survival post-axotomy may involve endoplasmic reticulum (ER) stress and the unfolded protein response (UPR), both of which are induced and activated after nerve damage (Penas et al., 2007; Ohri et al., 2011; Fan et al., 2015). If ER stress is activated, a number of chaperone proteins are upregulated such as GRP78/BiP (that co-chaperones with HSP70 or σ1R) and GRP94, an ER homolog of HSP90. These chaperones attempt to correct the stress by mediating proper protein folding and thus prevent cell death through induction of pro-survival factors such as Bcl2. If chaperones are unable to prevent accumulation of unfolded or misfolded proteins, the UPR is activated to minimize overloading by unfolded proteins. If however the stress is still unmanageable and homeostasis cannot be restored in a timely manner, apoptosis is induced (Fu and Gao, 2014). Enhancement of ER stress components such as CHOP, XBP1, and ATF6 has been observed after spinal cord contusion (Penas et al., 2007; Ohri et al., 2011), L5 spinal nerve ligation (Zhang et al., 2015) and sciatic nerve crush (Mantuano et al., 2011), with BiP enhancement observed in the latter two studies. The hypothesis is that the chaperones are attempting to correct the injurious processes and prevent cell death. However, very few studies have definitively linked chaperones with correcting ER stress and UPR activation after PNS and CNS axotomy. Penas et al. (2011b) have ventured into this area by noting that the balance between BiP and CHOP drives cell fate. As a result, they sought to modulate this ratio in favor of BiP using valproate (VPA). Although it did not augment BiP levels, high doses of VPA in a severe spinal cord contusion model reduced CHOP levels which correlated with reduced oligodendrocyte, myelin and axonal loss and better functional recovery. Of interest, the same lab has implicated chaperones in preventing another cell death process, autophagy, after axotomy. It was noted that autophagy markers such as BECLIN 1, LC3II, and LAMP-1 were enhanced in motor neurons after spinal root avulsion along with downregulation of BiP. The authors concluded that BiP decrease is a signature of the degenerating process, since its overexpression led to an increase in motor neuron survival (Penas et al., 2011b). However, this is correlative and more studies are needed to clearly clarify that the upregulation of chaperone proteins seen after PNS and CNS nerve damage is an attempt to correct ER stress or autophagy-induced cell death.

### Axon regeneration

In addition to cell survival, chaperones have been associated with regeneration of damaged PNS and CNS axons. For example, clusterin was found to be important for regrowth of sensory neurons after sciatic nerve transection and crush injury since this process was impaired in clusterin null mice (Wright et al., 2014). Further, exogenous application of the small heat shock protein, αBC, promoted neurite outgrowth of rat retinal cells (Wang et al., 2012). *In vivo*, the crystallin mediated regeneration of crushed rat optic nerve fibers through reduced activation of RhoA and phosphorylation of cofilin and myosin light chain. Other mechanisms by which chaperones mediate regeneration may involve modulation of axonal and Schwann cell cytoskeleton. Specifically, HSP27 was found to promote axonal outgrowth by possibly promoting assembly of intermediate filament proteins in Schwann cells (Hirata et al., 2003). In addition, Ma et al. (2011) noted that enhanced motor function recovery attributed to HSP27 was likely due to increased motor synapse reinnervation. To expand upon effects on functional recovery, exogenous application of αBC was discovered to promote locomoter recovery following spinal cord contusion injury in mice, with the improvement linked to reduced tissue damage and inflammation (Klopstein et al., 2012).

### Myelination

We recently showed that αBC contributes to remyelination in the PNS after sciatic nerve crush in mice (Lim et al., 2017). Specifically, the small HSP appears to modulate the conversion of de-differentiated Schwann cells back to a myelinating phenotype and that this occurs through a neuregulin 1 Type III/AKT/ErbB2 mechanism. A hint that the crystallin may be involved in myelination was demonstrated earlier by D'Antonio et al. (2006) who found a correlation between the expression of the HSP and formation of myelinating Schwann cells during development. αBC is not the only chaperone found to be involved in Schwann cell function after PNS injury. BAG1, which is enhanced in Schwann cells after sciatic nerve crush, was shown to be important for differentiation of myelinating Schwann cells since knockdown of BAG1 with siRNA reduced the number of protein zero positive Schwann cells in culture (Wu et al., 2013).

### Neuropathic pain

All of the functions discussed above are beneficial, but chaperones may be involved in promoting injury-related neuropathic pain. The chaperone σ1R, was found to be associated with neuropathic pain after sciatic nerve ligation since use of an antagonist called 4-[2-[[5-methyl-1-(2-naphthalenyl)-1H-pyrazol-3-yl]oxy]ethyl]morpholine) inhibited spinal sensitization and pain hypersensitivity (Romero et al., 2012). In corroboration, Pan et al. (2014) showed that σ1R was involved in pain sensitivity by sensory neurons in a rat sciatic nerve ligation model, since its antagonism with 1-[2-(3,4-dichlorophenyl)ethyl]-4-methylpiperazine dihydrochloride or *N*-[2-(3,4-dichlorophenyl)ethyl]-*N*-methyl-2-(dimethylamino)ethylamine dihydrobromide, reduced pain through restoration of calcium influx. Further, σ1R^−/−^ mice display reduced central sensitization and diminished hyperalgesic responses after sciatic nerve ligation (de la Puente et al., 2009). These results would suggest that the expression of σ1R would have to be increased or at least maintained from its non-injured level. However, Bangaru et al. (2013) showed that σ1R expression was reduced in DRG cells after sciatic nerve ligation. Whether there is differential expression in DRGs vs. axons after injury needs to be clarified especially knowing that local protein synthesis can occur within PNS axons (Court et al., 2011).

### Inflammation

There is a large literature on chaperones and inflammation. However, very little research has been conducted on how these proteins affect the immune response after PNS and CNS nerve injury. Klopstein et al. (2012) showed that αBC reduced inflammation in the mouse spinal cord after contusion injury. On the other hand, Fan et al. (2015) linked GRP78/BiP with necroptosis and ER stress in macrophages/microglia after mouse spinal cord contusion. Considering the beneficial role that the robust immune response plays after PNS axon damage, there is an opportunity to understand what function(s) chaperone proteins play in the protective PNS immune response as opposed to the slow, limited and seemingly detrimental inflammation seen after CNS axotomy.

Altogether, a number of chaperones play various beneficial roles after PNS and CNS injury including promoting neuronal survival, axon regeneration, remyelination, Schwann cell differentiation, and functional recovery. However, others such as σ1R appear to mediate less desirable effects such as neuropathic pain.

## Therapeutic strategies that involve chaperones

Because of the many reported beneficial functions of chaperone proteins after CNS and PNS nerve injury, some efforts have been made to harness their protective properties to enhance repair. Peroxisome proliferator-activated receptor-gamma (PPARγ) is a ligand-activated transcription factor of the nuclear hormone receptor superfamily whose agonist pioglotazone improved functional recovery and reduced motor neuron loss, astrogliosis, and microglial activation after rat spinal contusion injury. This improvement was attributed in part to the augmented expression of HSP27, HSP32, and HSP70 in the cord (McTigue et al., 2007). Using another PPARγ ligand, rosiglitazone, Yi et al. (2008) implicated chaperones in promoting survival of the damaged spinal neurons after brain contusion injury since neuronal survival correlated with enhanced expression of HSP27, HSP70 and HSP32. Along the same lines on neuronal survival, because σ1R agonists possess potent anti-apoptotic abilities, one of its agonists, 2-(4-morpholinethyl)1-phenylcyclohexanecarboxylate, was studied in the context of spinal root avulsion in the rat. Here, a marked increase in motor neuron survival was evident, which correlated with a decrease in astrogliosis and an increase in the σ1R co-chaperone, BiP. However, considering the neuropathic pain-inducing effects of σ1R described in the previous section, one would have to decipher how to achieve the benefits of neuronal survival without developing pathological effects such as pain.

With respect to other therapies involving chaperones, improvement in functional recovery in rats with a contusion spinal cord injury following interferon-beta 1b treatment was found to be related to enhanced HSP70 expression in the cord along with reduced polymorphonuclear leucocyte infiltration, hemorrhage, oedema and necrosis (Sengul et al., 2013). An interesting therapeutic intervention in spinal cord injury experiments has been the use of exercise in injured animals. Keeler et al. (2012) showed that functional recovery improved in animals that had been previously subjected to an exercise regiment. This benefit was associated with increased expression of HSP27 and HSP70 in the spinal cord. In the PNS, a similar effect was seen after chronic constriction of the rat sciatic nerve where exercise training attenuated neuropathic pain and which correlated with reduced inflammation and enhanced HSP27 expression (Chen et al., 2012).

Another chaperone-inducing agent that has been used as a therapy after nerve transection has been BRX-220 (bimoclomal analog). BMX-220 is a hydroxylamine derivative that promotes cell survival through increased expression of HSP70 and HSP90 (Vígh et al., 1997). In the CNS, BRX-220 improved motor neuron survival while enhancing HSP70 and HSP90 in the damaged spinal cord (Kalmar et al., 2002, 2003). Follow up studies in the PNS, showed that BRX-220 reduced pain sensations that correlated with enhanced HSP70 expression in sensory DRG neurons (Kalmar et al., 2003).

Finally, immunophilins are a group of proteins that serve as receptors for the immunosuppressant drugs cyclosporin A and FK506. Systemic administration of FK506 dose-dependently increased the rate of axonal regeneration and functional recovery in rats following a sciatic nerve crush injury. This was attributed to binding of FK506 to the immunophilins FKBP-12. However, it is possible that the beneficial function was through chaperone proteins—FK506 can also bind FKBP-52/FKBP-59, which has been identified as a heat shock protein (HSP56) and shown to enhance expression of HSP70 (Gold, 1997; Gold et al., 1997).

## Conclusion

Numerous studies over the past three decades have shown that the expression of chaperone proteins are not only altered following nerve damage to CNS and PNS neurons but that these proteins play an active role in the repair processes and in mediating some of the pathological events. Ongoing and future studies will have to consider how to harness the beneficial properties while reducing the injurious functions to enhance CNS and PNS recovery.

## Author contributions

SO wrote the manuscript with content discussion, editing, and figures design by AF and EL.

### Conflict of interest statement

The authors declare that the research was conducted in the absence of any commercial or financial relationships that could be construed as a potential conflict of interest.
